# Low-dose rituximab followed by mycophenolate mofetil for steroid-dependent/frequently relapsing nephrotic syndrome in children: a case series

**DOI:** 10.3389/fphar.2025.1646837

**Published:** 2025-08-26

**Authors:** Jide Song, Hong Chang, Yi Lin, Chunrong Shan, Jia Liu, Ranran Zhang, Nana Nie, Cui Bai, Shan Gao, Qiuye Zhang, Dahai Wang

**Affiliations:** ^1^ Department of Pediatric Nephrology, Rheumatology, and Immunology, The Affiliated Hospital of Qingdao University, Qingdao, China; ^2^ Department of Pediatrics, Qingdao Women and Children’s Hospital, Qingdao, China

**Keywords:** rituximab, mycophenolate mofetil, frequently relapsing nephrotic syndrome, steroid-dependent nephrotic syndrome, children

## Abstract

**Background:**

Rituximab (RTX) has gradually been accepted as a treatment for frequently relapsing nephrotic syndrome (FRNS) or steroid-dependent nephrotic syndrome (SDNS) in children, but no standardized recommendations for the dosage and combination therapy exist. Additionally, the efficacy and safety of low-dose RTX in FRNS/SDNS remain unclear, although it has been used to treat some autoimmune diseases.

**Methods:**

We report a case series of 24 children diagnosed with FRNS/SDNS treated with low-dose RTX followed by mycophenolate mofetil (MMF) for maintenance of remission of nephrotic syndrome between August 2021 and February 2023. These patients were followed up for at least 12 months.

**Results:**

The mean total dose for the initial four administrations of low-dose RTX was 470.83 ± 62.41 mg, which was significantly lower than the calculated values for one standard dose (525.62 ± 125.62 mg; *P* = 0.006) and two standard doses (1051.2 ± 251.23 mg; *P* < 0.001). After treatment initiation, the median follow-up was 24.6 (16.8, 28.5) months. At the 1-year follow-up, no child had experienced treatment failure, and the relapse-free rate was 83.3%. At the last follow-up, two children had experienced treatment failure, with both having frequent relapses, and the relapse-free rate was 75%. Compared with the calculated standard dose of RTX, low-dose RTX followed by MMF was less costly. No serious adverse reactions were observed during RTX use or follow-up, except for one death due to delayed treatment of severe infection.

**Conclusion:**

Low-dose RTX followed by MMF can extend the remission duration of FRNS/SDNS in children, and decrease the economic burden on families, while offering good safety.

## Introduction

Primary nephrotic syndrome (PNS) is one of the most prevalent glomerular diseases in the pediatric population (Londeree et al., 2022). Since the incidental discovery of a case of sustained remission of proteinuria in a child with nephrotic syndrome (NS) who was receiving rituximab (RTX)treatment for idiopathic thrombocytopenic purpura in 2004 (Benz et al., 2004), RTX has been gradually applied in the treatment of frequently relapsing nephrotic syndrome (FRNS) or steroid-dependent nephrotic syndrome (SDNS) in children. RTX has demonstrated substantial efficacy in the treatment of NS in children, both as a monotherapy and in combination with the immunosuppressive agent mycophenolate mofetil (MMF). Numerous case reports, case series studies, and randomized controlled trials have shown that treatment with RTX can allow discontinuation or reduction of steroid and/or immunosuppressant treatment and prolong the remission time in children with FRNS/SDNS, demonstrating good effects and good safety (Iijima et al., 2014; Kamei et al., 2017; Basu et al., 2018; Iijima et al., 2018; Iijima et al., 2022). As a result, the 2021 Kidney Disease Improving Global Outcomes (KDIGO) Glomerular Disease Treatment Guidelines and the 2023 International Pediatric Nephrology Association (IPNA) SSNS Guidelines both recommend the use of RTX in the treatment of FRNS/SDNS in children (Kidney Disease: Improving Global Outcomes Glomerular Diseases Work Group, 2021; Trautmann et al., 2023).

However, no standardized recommendations have been established regarding the dosage and treatment course of RTX, or potential combination therapy involving RTX, for PNS management. RTX is a biological agent and is expensive. Thus, long-term high-dose use will pose a serious financial burden on patients and their families. Accordingly, a treatment plan that takes both efficacy and economics into consideration is urgently needed. Current studies have confirmed that low-dose RTX offers good efficacy and safety for neuromyelitis optica spectrum disorder (NMOSD) and primary immune thrombocytopenia (ITP) (Yang et al., 2018; Li et al., 2019). We referred to these studies for NMOSD and ITP, low-dose RTX was defined as a first dose of 100 mg (weight <30 kg) or 200 mg (weight ≥30 kg), followed by the next three doses also all of 100 mg. However, whether low-dose RTX can provide the same efficacy and safety in the treatment of FRNS/SDNS in children has not been confirmed. Therefore, we hypothesized that the addition of MMF could consolidate the effect of low-dose RTX, allowing for a reduction of the RTX dose and decreased medical expenses. As a test of this hypothesis, the present case series study investigated the safety and efficacy of low-dose RTX followed by MMF in the treatment of FRNS/SDNS and examined the associated medical drug cost advantage to provide a reference for the clinical application of low-dose RTX in the treatment of FRNS/SDNS in children.

## Materials and methods

### Data collection and definitions

Our center has tried low-dose RTX therapy for treatment of NS in clinical practice, drawing on the usage of low-dose RTX in NMOSD and ITP. Therefore, the research team collected the clinical data of patients diagnosed with pediatric FRNS/SDNS and treated with low-dose RTX followed by MMF in the Affiliated Hospital of Qingdao University from August 2021 to February 2023 for retrospective analysis.

Certain concepts in this article are defined as follows, based on the IPNA Guide (Trautmann et al., 2023).

1. NS: Proteinuria that reaches the nephrotic criteria, combined with hypoalbuminemia (serum albumin <30 g/L).
2. SSNS: Complete remission within 4 weeks of treatment with prednisone or prednisolone (2 mg/kg/day or 60 mg/m^2^/day, maximum 60 mg/day).
3. SDNS: A case of SSNS with two consecutive relapses during recommended prednisone therapy for first presentation or relapse or within 14 days of discontinuation of prednisone therapy.
4. FRNS: Relapse ≥2 times within 6 months after the first remission or ≥3 times in any 12 months during the course of the disease.
5. Relapse: Urine dipstick ≥3 + (≥300 mg/dL) or urine protein creatinine ratio (UPCR) ≥200 mg/mmol (≥2 mg/mg) on a spot urine sample on 3 consecutive days in a child who had previously achieved complete remission.
6. Treatment failure: Development of frequent relapses, steroid dependence, or steroid resistance.
7. B-cell depletion: Peripheral blood B cells are eliminated, with the number of CD19+/CD20+ B cells in peripheral blood <5/µL or the proportion of total lymphocytes <1%.
8. B-cell reconstitution: The number of CD19+/CD20+ B cells in peripheral blood increases to >5/µL or accounts for >1% of total lymphocytes.
9. Body surface area (BSA) (Yu et al., 2003): BSA = 0.015925 (height (cm) × weight (kg))^1/2^.
10. Standard dose of RTX: 375 mg/BSA/dose.

### Case inclusion criteria

1. Onset age >1 year and <18 years.
2. Diagnosis of FRNS/SDNS.
3. Still steroid-sensitive during the last relapse.
4. Percentage of CD19^+^ cells in lymphocytes >1%.

### Case exclusion criteria

1. Previous monoclonal antibody treatment.
2. Concurrent infection necessitating hospitalization, such as tuberculosis, hepatitis, HIV, *etc.*
3. History of autoimmune disease.
4. History of tumor.

### Treatment programs

1. RTX: 1 dose per week for 4 consecutive weeks, with a total of four doses. The first dose is 100 mg (weight <30 kg) or 200 mg (weight ≥30 kg), and the next three doses are all 100 mg. If the percentage of CD19^+^ B cells in peripheral blood is >1%, an extra dose of RTX equivalent to the first dose was given. During the disease, extra doses of RXT could be administered multiple times. However, the interval between the first and the last dose should not exceed 2 years.
2. MMF: Oral MMF was initiated 1 week after the fourth dose of RTX, at a dosage of 20–30 mg/kg/day, administered every 12 h, with a maximum single dose ≤1 g. In cases in which MMF had been administered prior to the use of RTX, it was recommended to discontinue MMF before the RTX infusion. The treatment course was at least 1 year.
3. Glucocorticoids: Empirical reduction.

### Primary outcome

The primary outcomes measured were the period of no treatment failure (no frequent relapses and no steroid dependence).

### Secondary outcomes

Secondary outcomes included the time of the first relapse, the occurrence of any relapses, the total number of relapses, the occurrence of frequent relapses, and the occurrence of adverse events. The percentage of CD19^+^ B cells was monitored, and the total medical costs (including outpatient and hospitalization expenses at our center) for low-dose RTX and MMF within 6 months were calculated. Indirect costs were not calculated due to the lack of comprehensive data about costs incurred outside our hospital.

### Statistical analysis

Statistical analysis was performed using SPSS 26.0 software. The Shapiro–Wilk normality test was performed for measurement data. Continuous variables with a normal distribution are presented as mean ± standard deviation (SD); non-normal variables are reported as median (interquartile range). A paired sample *t*-test was used for data comparison. Count data are expressed as percentages. The duration of sustained remission of proteinuria during the observation period from the date of the first dose of RTX was analyzed using the Kaplan-Meier method. All comparisons are two-tailed, with values of *P* < 0.05 considered statistically significant. Our study was approved by the Ethics Committee (EC) of the Affiliated Hospital of Qingdao University (QYFYWZLL29344) and adhered to the principles outlined in the Declaration of Helsinki. Due to the retrospective nature of the study and the use of unidentified data, the EC waived the need for informed consent from patients for inclusion in this study.

## Results

### Clinical characteristics

The study cohort comprised 24 children, including 18 boys and 6 girls, with a mean age of 11.6 ± 3.4 years and a disease course of 65 (45, 108) months. Among these cases, 11 patients underwent renal biopsy, of whom 10 had minimal change disease and 1 had focal segmental glomerulosclerosis. The median steroid dose before RTX treatment was 20 (10, 50) mg, and the average percentage of CD19^+^ B cells was 11.9% ± 7%. The median follow-up time in this study was 24.6 (16.8, 28.5) months, and the longest follow-up time was 33 months. Additional clinical characteristics of the patients are presented in Table 1.

**TABLE 1:** Baseline clinical characteristics of 24 FRNS/SDNS pediatric patients.

Variable	Value
No. of patients	24
Age, years	11.6 ± 3.4
Disease duration, months	65 (45, 108)
Gender	
Female	6 (25%)
Male	18 (75%)
Serum creatinine, mg/dL	32.0 ± 8.5
eGFR, mL/min/1.73 m^2^	173 ± 37
Albumin, g/L	33.1 ± 7.9
Total cholesterol, mmol/L	6.5 ± 2.6
Percentage of CD19^+^ B cells, %	11.9 ± 7.0
Pathology	11 (45.8%)
Minimal change disease	10 (91%)
Focal segmental glomerulosclerosis	1 (9%)
Glucocorticoid, mg	20 (10, 50)
Concomitant medication	
ACEI or ARB	13 (54%)
MMF	4 (16.7%)
CTX	11 (45.8%)
Tacrolimus	0 (0%)
Disease severity	
FRNS/SDNS	17 (71%)
Complicated FRNS/SDNS	7 (29%)

Data are presented as median (interquartile range), mean ± standard deviation, or n (%). Abbreviations: eGFR, estimated glomerular filtration rate; ACEI, angiotensin-converting enzyme inhibitor; ARB, angiotensin receptor blocker; MMF, mycophenolate mofetil; CTX, cyclophosphamide; FRNS, frequently relapsing nephrotic syndrome; SDNS, steroid-dependent nephrotic syndrome.

### Drug dosage

**FIGURE 1:**
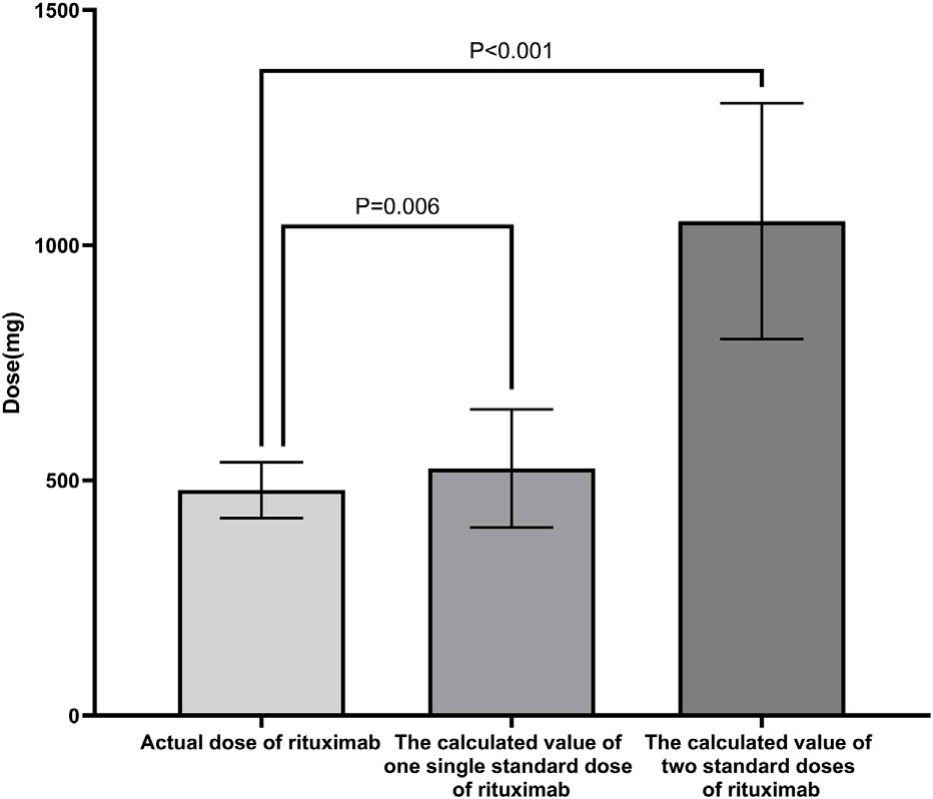
Comparison of RTX dosages in different schemes. The mean total dose of the initial four administrations of low-dose RTX was significantly lower than the calculated values for one standard dose and two standard doses of RTX. Standard dose of RTX: 375 mg/BSA/dose. Abbreviations: RTX, rituximab; BSA, body surface area.

The mean total dose of the initial four administrations of low-dose RTX therapy was 470.83 ± 62.41 mg (344.67 ± 71.26 mg/BSA), which was significantly lower than the calculated values for one standard dose (525.62 ± 125.62 mg; *P* = 0.006) and two standard doses (1051.2 ± 251.23 mg; *P* < 0.001) (Figure 1).

### Treatment failure

**FIGURE 2:**
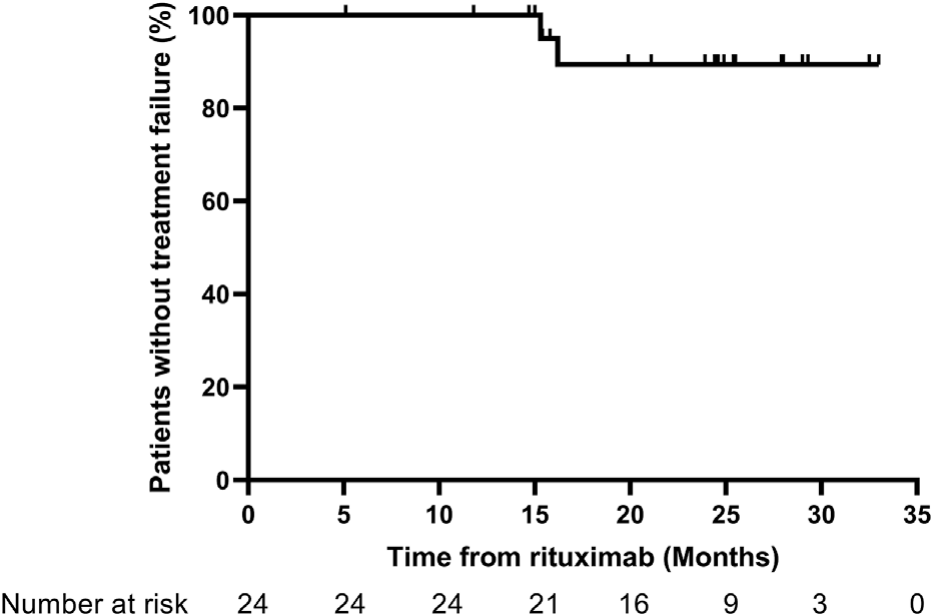
Kaplan–Meier curves for treatment failure. Treatment failure: development of frequent relapses, steroid dependence, or resistance.

Through the 1-year follow-up, none of the patients experienced treatment failure. At the last follow-up, 2 patients had experienced treatment failure and developed frequent relapses at 15.3 and 16.2 months after RTX therapy. The corresponding treatment failure rate was 8.3% in this study. The Kaplan–Meier curves for treatment failure are shown in Figure 2.

### Relapse

**FIGURE 3:**
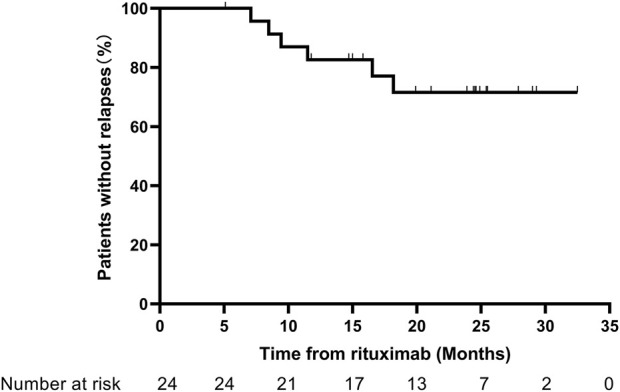
Kaplan–Meier curves for relapses.

The relapse-free rate within 1 year reached 83.3%, and the relapse-free rate at the last follow-up was 75%. Among the six cases that experienced relapse, the first relapse episodes occurred at 8.5, 7.1, 9.4, 18.2, 6.5, and 11.5 months after RTX. Two cases showed B-cell exhaustion during the first relapse, and the remaining four cases experienced their first relapse after B-cell reconstitution. A total of 16 patients received additional RTX, at an interval of 9.3 (8.3, 12.3) months from the initial RTX treatment. The Kaplan–Meier curves for relapse are presented in Figure 3.

### CD19^+^ B cells

**FIGURE 4:**
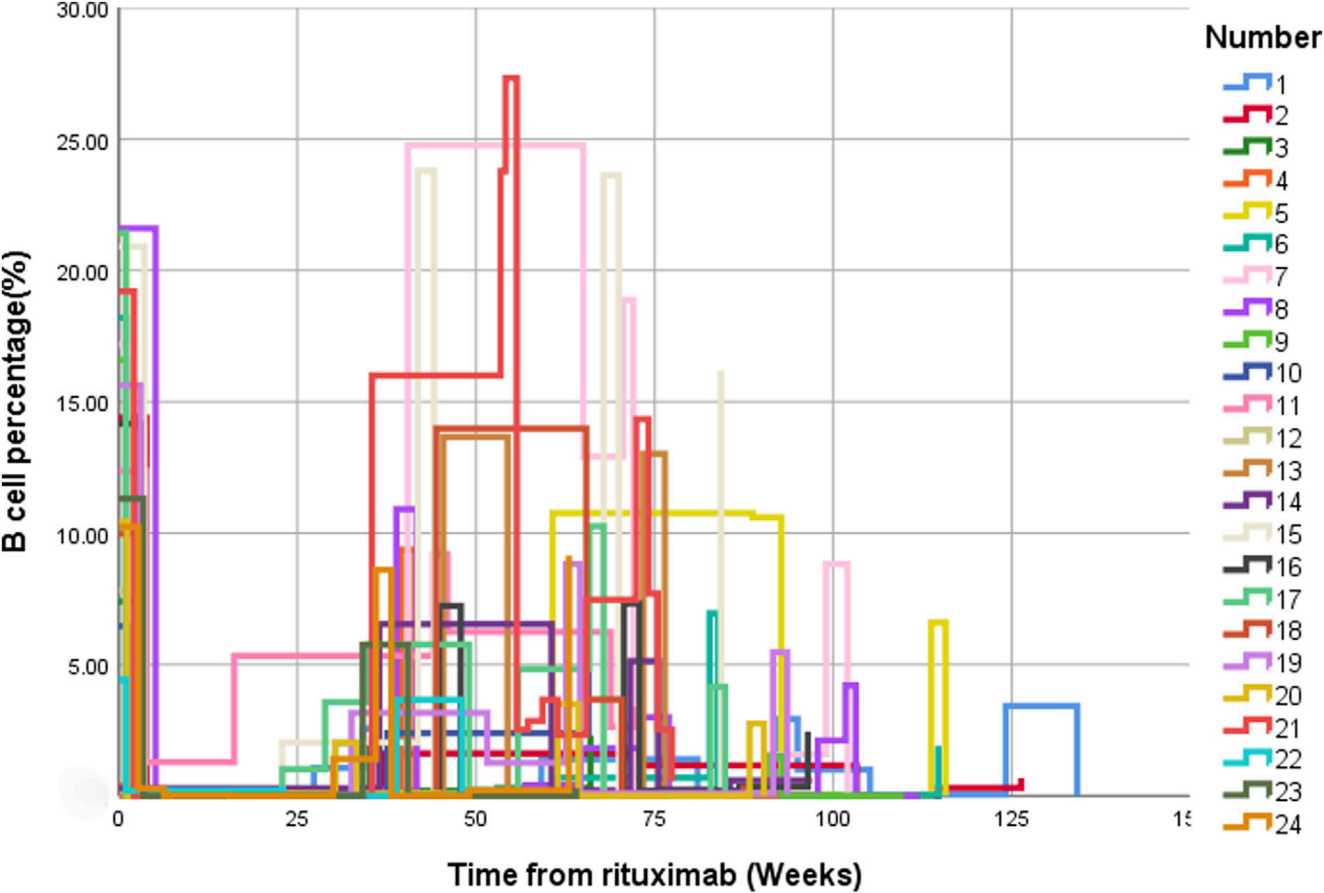
Changes in the percentage of CD19^+^ B cells before and after treatment with low-dose RTX. CD19^+^ B cell changes over time presented using multiple line graphs (interpolation: type [step], location [left]). Abbreviation: RTX, rituximab.

Following low-dose RTX treatment, significant decreases in the CD19^+^ B cell counts and proportions were observed (Figure 4). After B-cell depletion, the average time to first B-cell reconstitution was 8.8 (7.2, 9.9) months.

### Steroid treatment

At the last follow-up, the steroid dosage being taken by the 24 pediatric cases was lower than it had been before RTX therapy. Sixteen patients (66.6%) had been able to completely stop steroid therapy, while the other eight patients had not completely reduced or stopped steroid use due to relapse or repeated proteinuria.

### Medical costs

**FIGURE 5:**
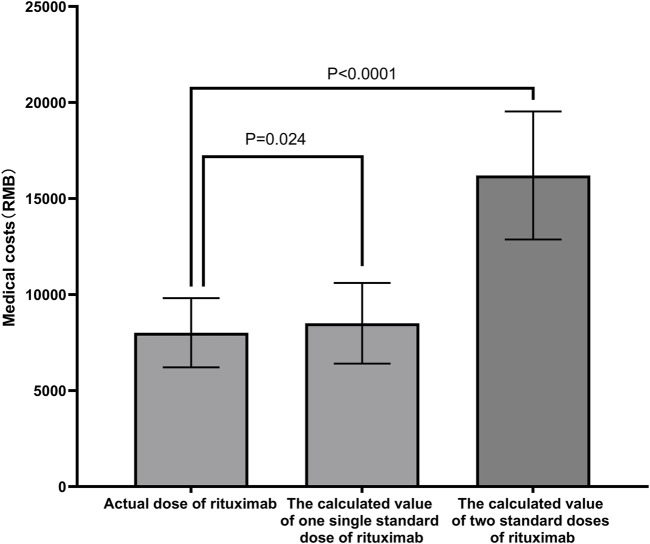
Clinical costs of different schemes. The medical costs of the low-dose RTX followed by MMF were significantly lower than the calculated costs of one or two standard doses of RTX. Abbreviations: RTX, rituximab; MMF, mycophenolate mofetil; RMB, Renminbi.

The medical costs for low-dose RTX followed by MMF were significantly lower than the calculated costs for one standard dose of RTX and two standard doses of RTX (Renminbi [RMB] 8,013 ± 1803 for low-dose RTX vs. RMB 8508 ± 2099 for one standard dose, *P* = 0.024, and RMB 16203 ± 3,380 for two standard doses, *P* < 0.001; Figure 5).

### Adverse events

The adverse events observed among the study population during follow-up included 39 infection events, 6 infusion reactions, 2 episodes of neutropenia, and 68 episodes of hypogammaglobulinemia (Table 2). Most adverse events were classified as grades 1 to 3, and only adverse events of grades 2 and above are reported here. The adverse reactions were relieved after supportive therapy such as anti-infective therapy and immunoglobulin infusion supportive treatment.

**TABLE 2:** Adverse events categorized by severity.

Adverse events	No. of adverse events (No. of patients)
Grade 2 adverse events	
Infusion related reaction	7 (4)
Allergic reaction	0 (0)
Upper respiratory infection	10 (7)
Bronchial infection	15 (7)
Gastroenteritis	1 (1)
Conjunctivitis	1 (1)
Urinary tract infection	3 (2)
Hypogammaglobulinemia	53 (12)
Neutropenia	2 (2)
Grade 3 adverse events	
Urinary tract infection	1 (1)
Pneumonia	7 (3)
Tuberculosis infection	1 (1)
Hypogammaglobulinemia	13 (5)
Agranulocytosis	0 (0)
Grade 4 adverse events	0 (0)
Grade 5 adverse events	
Death	1 (1)

One patient death was reported in this study. The deceased child was in a state of B-cell depletion after the initial treatment with low-dose RTX. During this period, although recommended, sulfamethoxazole was not administered to prevent infection. The child developed fever and cough and was brought to our outpatient clinic for treatment. After 4 days of intravenous ceftriaxone and azithromycin, the child did not have fever again but still had cough and dyspnea. The child’s parents did not seek medical treatment for these symptoms. One day later, the child was in poor spirits, and blood gas analysis showed respiratory failure. He was admitted to the pediatric intensive care unit in our hospital with a percentage of CD19^+^ B cells of 0% and immunoglobulin G level of 2.8 g/L. This patient’s fungal G result was 461.24 pg/mL (reference range <60 pg/mL), and aspergillus GM test result was 0.97 (reference range <0.5). Because the child was in an immunosuppressive state, the infection had progressed rapidly. The patient received active anti-infection treatment, ventilator respiratory support, closed chest drainage, and other treatments, but the child’s condition continued to progress, eventually leading to death from sepsis, pulmonary hemorrhage, diffuse intravascular coagulation, and multiple organ failure.

## Discussion

B-cell dysfunction is one of the important mechanisms of PNS (Colucci et al., 2022). RTX can act on the CD20 molecule on the surface of B cells to target and deplete B cells, thereby reducing the production of autoantibodies in order to treat PNS (Sinha et al., 2021). Numerous clinical controlled trials have demonstrated that RTX has a definitive therapeutic effect in the treatment of FRNS/SDNS, supporting prolonged remission and allowing for a reduction in steroid dosage (Iijima et al., 2014; Ruggenenti et al., 2014; Ravani et al., 2015; Iijima et al., 2022). MMF is an immunosuppressant that selectively blocks *de novo* purine synthesis, thereby suppressing T-cell and B-cell proliferation and antibody production. RTX combined with MMF has also been shown to be effective in treating FRNS/SDNS. Kazumoto et al. conducted a multicenter, randomized, double-blind, placebo-controlled trial and found that MMF maintenance therapy after RTX resulted in lower relapse rates and significantly lower daily steroid doses than RTX alone during the treatment period (Iijima et al., 2022). A study by Shuichi et al. also showed that the relapse rate among SDNS patients was significantly reduced with the use of MMF as maintenance therapy after RTX infusion (Ito et al., 2011).

In addition to standard doses, low-dose RTX has been used in the treatment of autoimmune diseases and has shown good efficacy and safety in NMOSD, ITP, anti-NMDAR (N-methyl-D-aspartate receptor) encephalitis, and others (Zaja et al., 2010; Wang et al., 2017; Yang et al., 2018). In the Chinese guideline on the diagnosis and management of adult ITP (version 2020), standard dose and low-dose RTX are equally recommended as second-line treatments for ITP (Thrombosis and Hemostasis Group, 2020). A retrospective analysis of NMOSD patients treated with RTX showed that ultralow dose RTX (100–300 mg) was noninferior to low-dose RTX (500 mg) in preventing NMOSD relapses (Zhang et al., 2024). However, the use of low-dose RTX to treat NS has been rarely reported. One previous study suggested that low-dose RTX (200 mg per week × 4 followed by 200 mg every 6 months) could significantly reduce the relapse rate and steroid dose in adults with minimal change disease and fewer side effects (Zhang et al., 2023). In a retrospective single-arm cohort study, 13 adult patients received low-dose RTX therapy (<375 mg/m^2^ BSA), and the median complete remission maintenance period was 25 months (Fujimoto et al., 2021). The use of low-dose RTX in pediatric cases of NS has not been reported previously though, making this the first study to elucidate the efficacy and safety of low-dose RTX followed by MMF for childhood-onset FRNS/SDNS.

Low-dose RTX can effectively achieve B lymphocyte depletion and also eliminate CD19^+^ B cells. A previous study showed that low-dose RTX can eliminate CD19^+^ B cells in the treatment of NMOSD (Yang et al., 2018). Another study showed that in patients with anti-NMDAR encephalitis who did not respond to first-line immunotherapy, early application of lower dosages of RTX could efficiently reduce CD19^+^ B cell counts in peripheral blood, and the median B-cell reconstruction time was 25 weeks (Wang et al., 2017). The same result was observed in ITP, showing that low-dose RTX is effective in ITP, with B cell counts remaining significantly reduced after 6 months (Zaja et al., 2010). The present study observed similar results after treatment with four low doses of RTX. Although the dosage was significantly lower than that of one standard dose of RTX, B-cell depletion occurred in all patients, and the median B lymphocyte reconstruction time was 8.8 (7.2, 9.9) months.

In this self-controlled before and after study all 24 pediatric cases of FRNS/SDNS treated with low-dose RTX followed by MMF changed status from frequent recurrence/steroid dependence to infrequent relapse or non-recurrence within 1 year. No case experienced treatment failure within 1 year, and only 16.7% of cases experienced recurrence. During the entire follow-up period, only 8.3% of cases experienced treatment failure, and 25% of cases experienced relapse. A previous multicenter, double-blind, randomized, placebo-controlled trial showed that the median relapse-free period was significantly longer in their RTX-treated group (four doses of standard-dose RTX) than in their placebo group (267 days vs. 101 days) (Iijima et al., 2014). Another trial of MMF after four doses of standard-dose RTX for FRNS/SDNS showed that 15.4% patients in the MMF group presented with treatment failure, compared with 53.8% in the placebo group (Iijima et al., 2022). A randomized clinical trial of Tacrolimus or MMF for FRNS/SDNS showed that the mean (SD) time to first relapse was significantly longer in the Tacrolimus group (323.99 [98.33] days) than in the MMF group (263.21 [132.84] days), and the Tacrolimus group showed a lower annual relapse rate than the MMF group (17.78% vs. 41.48%) (Wang et al., 2025). These collective results suggest that low-dose RTX followed by MMF also has a role in maintaining disease remission in FRNS/SDNS. Thus, the combination of low-dose RTX and MMF can be used as a two-pronged treatment method to treat pediatric patients with FRNS/SDNS and achieve relatively satisfactory results.

One strategy for maintaining continued remission of NS is to monitor B lymphocyte responses and repeat RTX treatment if B lymphocyte recovery occurs. Sellier-Leclerc et al. reported that after 15 months of B lymphocyte depletion, 60% of patients achieved long-term remission through multiple courses of RTX (Sellier-Leclerc et al., 2012). An international study showed that pediatric patients who received repeated courses of RTX for FRNS/SDNS experienced improved clinical responses and acceptable side effects (Chan et al., 2022). In the present study, the first re-administration of low-dose RTX treatment was given after 9.3 (8.3, 12.3) months. Four patients relapsed after B-cell reconstruction, but after further treatment with low-dose RTX, B cells were exhausted again, leading to NS maintenance remission and indicating a good treatment effect. Through the repeated use of low-dose RTX and continuous MMF therapy, 87% of the patients experienced sustained remission of urine protein over 1 year and none had frequent relapses. Over the entire follow-up period, only 6 cases relapsed, of which 2 had frequent relapses. This self-controlled before and after study showed that repeated administration of low-dose RTX and continuous MMF therapy can maintain urine protein remission and a relapse-free state for an extended period.

NS requires long-term medication therapy, and multiple relapses lead to high hospitalization costs, which pose a heavy burden for most families. Accordingly, less expensive treatment solutions that can maintain the relapse-free condition are urgently needed. A previous clinical study found that among 18 pediatric patients with SDNS treated with calcineurin inhibitors (CNIs) or RTX, the cost of RTX was comparable to that of CNIs, but the number of clinical visits was less, which may reduce the medical burden on patients’ families (Iorember et al., 2018). A retrospective study showed that although targeted drugs such as RTX are expensive, they significantly reduce the number of relapses and total medical costs (mainly hospitalization costs), indicating their clinical and cost benefits (Takura et al., 2018). In the present study, the medical costs of the low-dose RTX followed by MMF were lower than those of the calculated single and two standard-dose RTX. With lower costs, low-dose RTX combined with MMF treatment also may improve treatment compliance among children’s families while ensuring efficacy. In comparison with the standard dose RTX regimen, low-dose RTX combined with MMF was found to be equally effective at maintaining long-term remission in children with FRNS/SDNS while providing a more cost-effective alternative.

Although studies have reported that most patients with NS tolerate RTX well, some children may experience severe adverse events, such as anaphylactic shock, rhabdomyosarcoma, fulminant myocarditis, fatal viral infection, and neutropenia (Bayram et al., 2020; Kakleas et al., 2021; Zurowska et al., 2023). In our study, adverse events included infection (9 cases), infusion reaction (3 cases), immunoglobulin reduction (16 cases), and death (1 case). Except for the deceased patient, the adverse events among the other children were effectively controlled after symptomatic treatment, and no serious infection or damage to important organs was detected during the follow-up. Because treatment with RTX and MMF will inhibit the normal immune activity of B cells, SMZ should be used after the initial treatment to prevent possible serious infections. When infection occurs in these patients, they are prone to develop severe infections and should seek and receive immediate medical treatment. RTX treatment is generally believed to be safe, provided that the medication is taken according to the doctor’s instructions, with timely monitoring and regular review.

The shortcomings of the present study are that this was a retrospective case series study and lacked a control group, and the results could be affected by inconsistent follow-up times, missing data, and sample selection bias. Our center is conducting a case-control study on the treatment of FRNS/SDNS with different doses of RTX, which involves a larger patient population. We also plan to conduct a multicenter randomized controlled trial involving more nephrology centers in the future to ensure the diversity of the patient population. Determination of the optimal administration regimen for low-dose RTX still requires more large-scale randomized controlled trials.

## Conclusion

In summary, this is the first study to test the efficacy and safety of low-dose RTX followed by MMF for childhood-onset FRNS/SDNS. This treatment regimen was found to significantly reduce the relapse rate and extend the relapse-free time, with reduced treatment costs and good safety.

## Data Availability

The raw data supporting the conclusions of this article will be made available by the authors, without undue reservation.

## Abbreviations

RTX, Rituximab; FRNS, frequently relapsing nephrotic syndrome; SDNS, steroid-dependent nephrotic syndrome; MMF, mycophenolate mofetil; PNS, primary nephrotic syndrome; NS, nephrotic syndrome; SSNS, steroid-sensitive nephrotic syndrome; KDIGO, Kidney Disease Improving Global Outcomes; IPNA, International Pediatric Nephrology Association; NMOSD, neuromyelitis optica spectrum disorder; ITP, immune thrombocytopenia; UPCR, urine protein creatinine ratio; BSA, body surface area; SD, standard deviation; EC, Ethics Committee; RMB, renminbi; NMDAR, N-methyl-D-aspartate receptor; CNIs, calcineurin inhibitors; eGFR, estimated glomerular filtration rate; ACEI, angiotensin-converting enzyme inhibitor; ARB, angiotensin receptor blocker; CTX, cyclophosphamide.
